# Is group cognitive behaviour therapy for postnatal depression evidence-based practice? A systematic review

**DOI:** 10.1186/1471-244X-13-321

**Published:** 2013-11-28

**Authors:** Alison Scope, Joanna Leaviss, Eva Kaltenthaler, Glenys Parry, Paul Sutcliffe, Mike Bradburn, Anna Cantrell

**Affiliations:** 1 School of Health and Related Research (ScHARR), The University of Sheffield, 30 Regent Street, Sheffield S1 4DA, UK; 2 Division of Health Sciences, University of Warwick, Coventry CV4 7AL, UK

## Abstract

**Background:**

There is evidence that psychological therapies including cognitive behaviour therapy (CBT) may be effective in reducing postnatal depression (PND) when offered to individuals. In clinical practice, this is also implemented in a group therapy format, which, although not recommended in guidelines, is seen as a cost-effective alternative. To consider the extent to which group methods can be seen as evidence-based, we systematically review and synthesise the evidence for the efficacy of group CBT compared to currently used packages of care for women with PND, and we discuss further factors which may contribute to clinician confidence in implementing an intervention.

**Methods:**

Seventeen electronic databases were searched. All full papers were read by two reviewers and a third reviewer was consulted in the event of a disagreement on inclusion. Selected studies were quality assessed, using the Cochrane Risk of Bias Tool, were data extracted by two reviewers using a standardised data extraction form and statistically synthesised where appropriate using the fixed-effect inverse-variance method.

**Results:**

Seven studies met the inclusion criteria. Meta-analyses showed group CBT to be effective in reducing depression compared to routine primary care, usual care or waiting list groups. A pooled effect size of d = 0.57 (95% CI 0.34 to 0.80, p < 0.001) was observed at 10–13 weeks post-randomisation, reducing to d = 0.28 (95% CI 0.03 to 0.53, p = 0.025) at 6 months. The non-randomised comparisons against waiting list controls at 10–13 weeks was associated with a larger effect size of d = 0.94 (95% CI 0.42 to 1.47, p < 0.001). However due to the limitations of the available data, such as ill-specified definitions of the CBT component of the group programmes, these results should be interpreted with caution.

**Conclusions:**

Although the evidence available is limited, group CBT was shown to be effective. We argue, therefore, that there is sufficient evidence to implement group CBT, conditional upon routinely collected outcomes being benchmarked against those obtained in trials of individual CBT, and with other important factors such as patient preference, clinical experience, and information from the local context taken into account when making the treatment decision.

## Background

Postnatal depression (PND) is defined as a non-psychotic depressive episode meeting standardised diagnostic criteria for a minor or major depressive disorder beginning in or extending into the postnatal period [1]. It is a major health issue for the affected individual and represents a significant risk to her child. PND has a substantial impact on the mother and her partner [2] and the family [3]. It may result in impaired maternal-infant interactions, [4] can lead to attachment insecurity, [5] impaired cognitive [6] and social-emotional development [7].

Prevalence estimates of 14.5% for developed countries[8] and 13% for developing countries [9] make PND an important global issue, although many cases may go undetected [10]. PND usually develops within the first three postnatal months, [11] with a peak incidence at around 4–6 weeks [1]. Although one study showed that most episodes last around three months and resolve spontaneously without treatment [11] another study demonstrated the presence of depression lasted over six months in over 50% of the sample, and in some cases depression was still present at four years [12].

The current National Institute for Health and Clinical Excellence (NICE) clinical guideline for antenatal and postnatal mental health [13] outlines the recommended care pathway to identify and treat women with PND, [13] although services vary widely across the UK. NICE guidance recommends psychological interventions such as individual cognitive behaviour therapy (CBT) or interpersonal therapy (IPT) for women with PND. However, the difficulty for practitioners in offering psychological interventions is that a number of potentially useful treatments may not meet the requirements of evidence-based practice (EBP) if it is applied in its strictest sense. The case example used here is whether it is effective to offer CBT in a group format.

Evidence-based practice (EBP) is defined as 'the conscientious, explicit, and judicious use of current best evidence in making decisions about the care of individual patients. The practice of evidence based medicine means integrating individual clinical expertise with the best available external clinical evidence from systematic research’ [14]. However, in some instances psychological therapies struggle to meet the demands of EBP as psychological and social aspects of treatments are often not taken into account [15]. The narrow ’single diagnosis, single intervention’ approach to evidence-based practice is in contrast with the broader appraisal clinicians need to make regarding psychological therapies. Factors other than the specific therapy method (e.g. CBT, IPT or psychodynamic) influence clinical outcomes, including the patient and the therapist forming a good working relationship, treatment length, patient demographics, patient preference, the skill level of therapist, and patient characteristics [16]. Medical Research Council (MRC) guidelines on trials for complex interventions recommend the need to look at the 'practical effectiveness’ of interventions, i.e. whether an intervention works in everyday practice [17]. Guidelines, even when supported by the best implementation practices, need to be supplemented by other clinical support methods and with methods of monitoring what is actually done in practice and its impact on the patient [18]. This includes exploring how effects may vary among recipients, and how this variation may be explained. Verkerk et al. [19] highlight the variability in the reported effectiveness of psychological interventions designed to reduce the risk of PND. They suggest that this variability may in part be explained by patient-related factors such as personality or patient preference for psychosocial intervention aimed at improving postnatal psychological adjustment. Further consideration of this is provided by Scope et al. [20]. This was a report of a systematic review that aimed to synthesise qualitative evidence relating to women’s perceptions and experiences of group cognitive behaviour therapy and other group interventions for postnatal depression. This study showed that women have contrasting experience of such treatments and that attention should be given to selecting patients for whom group treatment is most likely to be beneficial.

As there is an urgent need for effective treatments to prevent the poor outcomes of PND [10] it is important to assess potentially useful treatments in this broader context. While antidepressant medication has been validated as an effective treatment for general depression, mothers who are breastfeeding are often reluctant to take medication due to possible transmission of unwanted effects through breast milk [21]. Similarly, in a randomized controlled trial (RCT) comparing antidepressant medication (Fluoxetine) with cognitive-behavioural counselling in women with PND [22] the main reason for non-participation in the study was a reluctance to take antidepressant medication. It therefore seems imperative that non-pharmacological interventions are developed and evaluated and consensus is obtained about them. CBT is widely used for the treatment of PND and evidence supports its use in the treatment of mild to moderate non-childbirth related depression [23].

A large range of treatment trials, some involving group treatment, have been reported in earlier systematic reviews of women with PND [24,25]. A total of 21 studies were extracted including IPT, CBT, peer and partner support, infant sleep interventions, relaxation/massage therapy, maternal exercise, nondirective counselling, and infant-mother relationship therapy [24]. Overall no definite conclusions were reached about the relative effectiveness of many non-biological treatment approaches due to the lack of high quality investigations. In a review of psychosocial and psychological interventions for treating postpartum depression, Dennis and Hodnett [26] concluded that individual-based strategies were effective in decreasing depressive symptomatology. However, only one included study examined group-based interventions, therefore no conclusions could be drawn regarding this mode of delivery.

Although psychological interventions such as CBT are recommended for PND, access to individual CBT may be limited and group CBT treatment may be a potential alternative. This has the potential to reduce cost, therapist time, waiting time and to increase the number of available places. Patients are treated in groups of around eight people and treatment usually runs for 12 weeks often preceded by one individual session. There is little available evidence on the service provision of group CBT specifically for PND. There were two objectives of this study, the first was to systematically review and synthesise the evidence for effectiveness of group CBT compared to currently used package of care for women with PND, as measured by change in depression, and the second was to discuss other factors which may contribute to clinician confidence in implementing an intervention.

## Methods

This paper is an update of a review published as part of an HTA monograph. It adds to the findings reported in the monograph by updating the searches and conducting meta-analyses of the available evidence. A full description of the sources searched and more detailed information on excluded papers is described in Stevenson 2010 [27]. The search strategy was developed, and the search conducted, by an information specialist (AC). The searches aimed to identify all references relating to the clinical effectiveness of group CBT for PND. Seventeen electronic bibliographic databases were searched including: Medline, CINAHL, Cochrane, Embase and PsycINFO. The reference lists of relevant articles were checked and various health services related resources were consulted via the Internet. Grey literature searches were also undertaken using sources such as dissertation abstract databases. Population search terms included: depression, postpartum, postnatal depression, and post pregnancy depression. Searches were not restricted by intervention due to the complexity of defining the intervention and to prevent omission of relevant references. Searches were not restricted by language but non-English language papers were excluded at the sifting stage. Searches were undertaken in January 2008 and databases searched from 1950–2008. An update search, replicating the original search, was performed in March 2011. Databases were searched from 2008 to March 2011.

We included studies with populations that included women meeting the criteria of a standardized PND diagnosis using the diagnostic and statistical manual (DSM-IV) [28] or were screened for PND using the EPDS. If a clinical diagnosis is given, DSM-IV criteria for depressive disorder were used. DSM-IV recognises PND as a form of general depression with a specifier coded 'postpartum depression’ if it occurs within four week after giving birth. Some studies reported other measures of PND in addition to DSM-IV or EPDS. However, DSM-IV diagnosis or PND identified using the EPDS are the usual entry criteria for treatment programmes. Prenatal women, women with other comorbid psychiatric disorders or major medical problems or women who had been involved in a previous psychological programme were excluded. All interventions with elements deriving from cognitive behavioural principles were included, including those which were “psycho-educational” and in a group. All settings were included and all comparators considered including routine primary care, waiting list, individual CBT, group based counselling, medication, group behaviour therapy and group IPT. Studies were included if they reported depression measured using the Edinburgh Postnatal Depression Scale (EPDS) [29] or the Beck Depression Inventory (BDI) [30]. The EPDS is the most widely used self-report scale for the identification of PND. Whilst the EPDS was developed specifically to measure PND, PND may also be measured using the BDI which is a general population depression scale.

Papers were assessed according to the accepted hierarchy of evidence with systematic reviews of RCTs taken as the most authoritative forms of evidence and uncontrolled observational studies the least authoritative [31].

All full papers were read and quality assessed by two reviewers and a third reviewer was consulted in the event of a disagreement on inclusion or quality. Included papers were quality assessed using the Cochrane Risk of Bias Tool [32]. The quality elements addressed included selection bias (randomisation), reporting bias (outcome measures), attrition bias (ITT analysis), and detection bias (blinding of assessors). All data from included studies were extracted by two reviewers using a standardised data extraction form. Both RCTs and non-RCTs were considered for data synthesis. Data relating to each study’s key characteristics (e.g. population, intervention type and duration) and pertinent to the research question (i.e. clinical effectiveness) were extracted. The main outcome measure of interest was self-reported depression following intervention using either the BDI or the (EPDS).

Comparisons were quantified by standardised mean differences (SMDs) in which the effect size are presented in units of the standard deviation [33]. Statistical heterogeneity was quantified by the I^2^ statistic and formally tested by Cochran’s Q statistic. Where appropriate, studies were combined using the fixed-effect inverse-variance method. All analyses were undertaken using the metan [34] command within the Stata statistical package.

## Results

From the original searches we screened 7633 references and assessed the text of 153 full papers. The update search produced 2547 references which were screened and the text of 56 full papers was assessed. The study selection and exclusion process are summarised in Additional file 1: Figure S1. Six studies met the inclusion criteria from the original search, and the update search produced one additional study. The overall quality of the studies was low with a risk of bias in a number of domains, particularly for the non-RCTs as would be expected. Only two studies (both non-RCTs) reported reasons for loss to follow-up, only two studies reported using a power calculation, and only two reported that the outcome assessor had been blinded. Furthermore one study did not include all study participants in their analyses.

The studies all included a group programme which incorporated some level of CBT theory or technique, although the degree of incorporation of CBT theory or technique varied markedly between studies. Three studies [35-37] specifically refer to at least a CBT component which appears to be a core-pre-defined aspect of the treatment. This however cannot be claimed with certainty for the Highet and Drummond [35] study due to a lack of detail in the report. The definitions in the other four included studies [38-41] are somewhat ill-specified and it is unclear whether CBT is a core aspect of the group treatment.

The study characteristics of the seven included studies are described in Table 1. The key components of study quality assessment are listed in Additional file 1: Figures S2 and S3.

**Table 1:** Summary of study characteristics for the seven included studies

**First author, date (country)**	**N**	**Design**	**Intervention**	**Concurrent therapy**	**Comparator**	**Number/duration of sessions**	**Measure/ timescale**	**Summary of main outcomes**
**Rojas 2007 (Chile)**	I: 114	RCT	Multi-Component Intervention including psychoeducational group and structured pharmacology if needed	Yes	Usual Care	8 x 50 minute weekly sessions	EPDS	Greater improvement in EPDS over three months in the intervention group, with differences between groups remaining at six months, although decreased
	C: 116							
**Milgrom 2005 (Australia)**	I: 46	RCT	Group-based Cognitive-Behavioural Therapy. Including psychoeducation, role-playing and discussion	N/R	C1: Group-based counselling	9 x 90 minute weekly sessions	BDI	Significantly greater reduction in depression scores after all interventions compared to routine primary care
	C1: 47				C2: Individual Counselling			
	C2: 66				C3: Routine Primary Care			
	C3: 33							
**Honey 2002 (UK)**	I: 23	RCT	Controlled Psychoeducational Group (PEG). Educational information on post-natal depression, strategies for coping, cognitive-behavioural techniques	N/R	Routine Primary Care	8 x 2 hour weekly sessions	EPDS	Significantly greater reduction in depressive symptoms in intervention group compared to routine primary care
	C: 22							
**Highet 2004 (Australia)**	I: 136	Non RCT	Eight different, not mutually exclusive, treatment groups	Yes	Waiting List	N/R	EPDS	Significant decrease in depression for those in intervention groups compared to those in the waiting list group
	C: 10							
**Clark 2003 (US)**	I: 13	Non RCT	Mother-Infant Therapy Group. Based on interpersonal, psychodynamic, family systems and cognitive-behavioural approaches	N/R	C1: Individual Interpersonal Therapy	12 x 90 minute weekly sessions	BDI	No significant difference in reduction of depressive symptoms for intervention group on BDI. No superiority of group therapy over individual therapy but both show greater improvement than control on CES-D
							CES-D	
	C1: 15				C2: Waiting List			
	C2: 11							
**Clark 2008 (US)**	I: 18	Non RCT	Mother-Infant Therapy Group. Interpersonal, psychodynamic, family systems, and cognitive behavioural approaches	N/R	Waiting List	12 x 120 minute weekly sessions	BDI	Significantly greater improvement in depressive symptoms for intervention group compared to waiting list group
	C: 14							
**Meager 1996 (Australia)**	I: 10	Non RCT	Group Treatment. Social and emotional support, education component, cognitive-behavioural component, networking and communication	Yes	Waiting List	10 x 90 minute weekly sessions	BDI	Significant improvement in depression in intervention group compared to waiting list group
							EPDS	
	C: 10							

*Abbreviations*: *RCT* Randomised Controlled Trial, *NR* Not reported, *BDI* Beck Depression Inventory, *EPDS* Edinburgh Postnatal Depression Scale, *I* Intervention, *C* Comparator.

Three of the included studies were RCTs [36,37,41] and four were non-randomised studies [35,38-40]. All seven studies had at least one comparison arm. Six of the studies compared group CBT to routine primary care or wait list group [36-41] although definitions of the interventions varied across studies. One non-RCT [35] compared group CBT to individual CBT and one [38] to IPT. One RCT [37] compared group CBT to group counselling and individual counselling.

### Study characteristics

Participants in the RCTs were recruited from community screening programmes of newly delivered mothers or referred by health visitors. For the non-RCTs recruitment was through health care provider referrals, newspaper advertisements and through advertisements in local hospitals and maternal and child health centres. One non-RCT [35] was a retrospective study of women who sought or had been referred for PND treatment.

### Study quality

Although the three RCTs reported the number of participants lost to follow-up, none provided the reasons for loss to follow-up. Two of the three non-RCTs reported the reasons for loss to follow-up, these included physical illness and difficulty in organising attendance [40] and not being contactable post-treatment, not considered to have PND by their healthcare provider, refusal to take part in the study, and stopping treatment prior to completion [37]. Follow-up exceeded 60% in all studies.

An acceptable method of randomisation was reported in all three RCTs. Although blinding of participants was not possible due to the nature of the intervention, two studies reported that the outcome assessor had been blinded [37,41] and two [37,41] reported power calculations. For the three non-randomised studies the study quality varied considerably. Participants included in the Meager and Milgrom [40] study were randomly assigned to either the group treatment or a wait-list group, but no randomisation method was reported. The Highet and Drummond [35] study examined patient records and no randomisation had taken place. In the Clark et al. [38,39] studies sequential assignment to group treatment or to the wait list was performed on the basis of matching for sociodemographic variables. None of the non-RCTs reported blinded assessment or a power calculation. Clark et al. [38] did not include all study participants in their analyses, with data only analysed for those participants with BDI scores of 16 or higher.

### Psychological outcomes

Three studies reported depression scores using the EPDS [35,36,42] and three reported BDI scores, [37-39] with the final study reporting scores for both scales [40]. For six of the seven studies the main outcomes related to improvement in depression symptoms. However, Clark et al. [38] reported infant development as the main outcome. Meta-analyses were conducted for depression outcomes Table 2.

**Table 2:** Psychological outcome results by measure and follow-up time

	**Intervention group**			**Control group**			
**Author**	**N**	**Mean**	**SD**	**N**	**Mean**	**SD**	**Comparison**
**BDI – 3 months**							
Milgrom (2005)	31	14.48	8.8	18	18.78	8.5	RCT v usual care
Clark (2003)	9	15.9	8.5	11	20.6	9.2	non-RCT v waiting list
Clark (2008)	18	12.42	7.08	14	20.5	7.27	non-RCT v waiting list
Meager (1996)	6	16.8	10	6	29.14	10	non-RCT v waiting list
**EPDS – 3 months**							
Rojas (2007)	101	8.5	6.41	108	12.8	6.92	RCT v usual care
Honey (2002)	23	14.87	5.97	22	16.95	5.44	RCT v usual care
**EPDS – 6 months**							
Rojas (2007)	106	10.9	6.67	102	12.5	6.92	RCT v usual care
Honey (2002)	23	12.55	4.62	22	15.63	7.28	RCT v usual care
Meager (1996)	6	15.8	7.6	6	28	7.6	non-RCT v waiting list

*Abbreviations*: *BDI* Beck Depression Inventory, EPDS Edinburgh Postnatal Depression Scale, RCT Randomised Controlled Trial.

Footnote: The total range of EPDS scores is 0–30. Scores within the range 12–30 suggest clinically significant depression. The total range of BDI scores is 0–63. Scores within the 1–9 range indicate minimal depression, 10–18 mild depression, 19–29 moderate depression, and 30–63 severe depression.

Means and standard deviations (SDs) were obtained directly from the papers where reported. In Rojas, [41] SDs were derived from 95% confidence intervals. Meager [40] did not report SDs, but these could be estimated from means and the p-value for their comparison.

Three studies, all RCTs, compared group CBT to usual care. Rojas [41] and Honey [36] assessed depression using the EPDS at 3 and 6 months post-randomisation, whereas Milgrom [37] used the BDI. Four non-randomised studies compared group CBT versus waiting list. Clark [38] and Clark [39] evaluated depression using BDI at 12 week post-randomisation, and Meager [40] used both BDI and EPDS to evaluate depression 10 weeks post-randomisation. The fourth study [35] was not considered for synthesis as treatment groups were not mutually exclusive.

**Figure 1:**
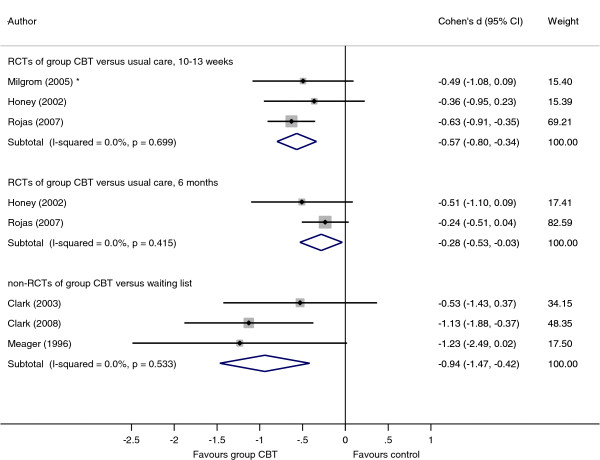
**Forest plot to show meta-analyses for three subgroups: i) group CBT versus usual care between 10–13 weeks; ii) group CBT versus usual care at 6 months; and iii) group CBT at versus waiting list between 10–13 weeks.** Footnote: Effect sizes in subgroups i) and ii) are calculated from EPDS scale with the exception of * which used BDI inventory. Effect sizes in subgroups iii) used BDI inventory.

These studies enabled meta-analyses to be performed among three subgroups: i) group CBT versus usual care between 10–13 weeks; ii) group CBT versus usual care at 6 months; and iii) group CBT versus waiting list between 10–13 weeks. The results are displayed graphically in Figure 1. In subgroup iii), Meager [40] was included using the BDI outcome data as this was the measure used by other studies in this subgroup, although results obtained using the EPDS were very similar. The I^2^ was equal to zero in all subgroups.

Group CBT was associated with improved outcomes in all three subgroups. A pooled effect size of d = 0.57 (95% CI 0.34 to 0.80, p < 0.001) was observed at 10–13 weeks post-randomisation, reducing to d = 0.28 (95% CI 0.03 to 0.53, p = 0.025) at 6 months. The non-randomised comparisons against waiting list controls at 10–13 weeks was associated with a larger effect size of d = 0.94 (95% CI 0.42 to 1.47, p < 0.001).

## Discussion

Seven comparative studies were identified in this review. Meta-analyses show that group CBT appeared to be clinically effective when compared to routine primary care, usual care or a waiting list group, although the reduction in depression scores was not consistent across time. These results should be interpreted with caution due to the limited number and quality of the studies. In addition, some of the included studies included concurrent therapy, the effects of which are difficult to separate from group treatment.

There was uncertainty as to how some of the described group treatments accurately reflect CBT and whether generalisations can be made due to participants being at different times postpartum in some studies. There is enough doubt in the quality, the level of CBT implemented in the group programmes, and the applicability to a PND population to limit any interpretations significantly. There is also debate over the comparability of the two measures used to evaluate PND, with some authors suggesting the generic BDI and PND-specific EPDS instruments are measuring intrinsically different features (e.g. Huffman, Lamour, Bryan, & Pederson, 1990; [43,44] Horowitz, Damato, Solon, Metzch, & Gill, 1995 [45]) Nonetheless, whilst the patient populations, CBT delivery and assessment tools may be very different, the outcomes observed in the meta-analyses were consistent with low I-squared values in all cases.

Although definitions of EBP acknowledge the importance of clinical expertise [14], and despite MRC recommendations for complex interventions to look at 'practical effectiveness’, the emphasis is still on RCTs and meta-analysis evidence. Patient preference factors together with clinical experience may be just as important in the treatment decision. The limitations of RCT evidence on psychological therapies in making treatment decisions have been extensively discussed [46,47]. These include the exclusion of patients with co-morbid diagnoses, mixed diagnoses, multiple or diagnoses which are not clearly defined and patients who drop-out of treatments. Further, reasons for such drop-outs and the data from patients who do drop out may be particularly important in the evaluation of psychological therapies. The quality of the primary studies included in this review, and the fact that only three RCTs have been reported, emphasise the problems of relying on RCT research evidence alone to make decisions about treatment of patients using psychological therapies. It has been argued that such evidence needs to be supplemented by practice-based evidence to yield a more robust, relevant, reliable, and comprehensive knowledge base [48].

One practical approach to this would be, where local circumstances make it unfeasible to offer individual therapies to all women with PND, to offer group methods whilst monitoring routine outcomes, which can then be benchmarked against the recovery rates obtained in trials of individual CBT. Such an approach raises logistic, methodological and ethical issues which we shall briefly consider here.

Increasingly, psychological service providers monitor outcomes of therapy routinely using standardised, patient-reported measures; typically either condition-specific measures or more generic or global measures which span well-being, psychological distress and functioning. The most commonly used measure for post-natal depression is EPDS; generic measures include CORE-OM and OQ-45. The two logistic challenges are a) ensuring that endpoint data are collected from all service users including those who leave before the end of therapy and b) that data are of sufficient quality for analysis. Data completeness in routinely collected datasets typically varies between sites; in one UK dataset of primary care psychological therapy and counselling services, pre-post completion rates varied between 3% and 99% for the poorest and best performing service, with an average of 39% (sd = 23) [49]. This demonstrates that it is possible with adequate care and attention to achieve very high levels of data completeness, but that this must be addressed vigorously. Ensuring data are of good quality again implies that local services pay close attention to how the measures are administered; for comparability with research-based data, it is preferable that questionnaires be administered outside the consulting room in a confidential setting.

Benchmarking service outcomes using group CBT against research trial reports using individual CBT should follow the method outlined by Minami et al., [50] which consists of three stages: constructing pre-post benchmarks from relevant clinical trials in terms of effect sizes or recovery rates, estimating the effectiveness of routine practice. again in terms of effect size or recovery rate, and comparing the routine practice effect size against the trials benchmark. Cohen’s rule of d = 0.2 is applied as the margin around the clinical benchmark. Some methodological and clinical issues surrounding this process are outlined by Lueger and Barkham [51]).

Ethical issues are raised by implementing a group therapy method without strong RCT evidence to support it, particularly where an individual approach may be more acceptable to patients. For example, in a comparison of the acceptability of group vs individual CBT for panic disorder and agoraphobia, Sharp, Power and Swanson [52] found that when given a free choice of group or individual CBT at the end of the waiting list period, the overwhelming majority (95%) of the waiting list patients chose individual CBT. It could therefore be argued that it is ethically more acceptable to offer group therapy as an alternative to no therapy, where individual CBT is not available to all women with PND.

Further research is needed to compare group CBT with routine primary care, which is likely to be in the main medication based with individual treatment and with other psychological therapies. In addition, particular aspects of group treatment require assessment such as the effect of the size of the group of patients, the duration of the sessions, the setting, the qualifications and optimal level of involvement of the facilitator. The review also highlights a need to assess the findings reported here in tandem with those reported on patient acceptability for this treatment [20] and for further research on the acceptability of group CBT to patients compared with alternative approaches.

## Conclusion

Although the evidence available is limited and of poor quality, we argue that, taken together with other important factors such as patient preference, there is sufficient evidence to implement group CBT conditional upon routinely collected outcomes being benchmarked against those obtained in trials of individual CBT.

## Competing interests

The authors declare that they have no competing interests.

## Author contributions

AS was the principle systematic reviewer, participated in the conception and design of the study and drafted the manuscript. JL participated in systematic reviewing, data analysis, and drafting the manuscript. EK, GP, and PS participated in the conception and design of the study and critically revising the manuscript. MB participated in data analysis. AC participated in the conception and design of the study and performed the literature searches. All authors read and approved the final manuscript.

## Pre-publication history

The pre-publication history for this paper can be accessed here:

http://www.biomedcentral.com/1471-244X/13/321/prepub

## Supplementary Material

Additional file 1: Figure S1 Summary of study selection and exclusion. **Figure S2.** Risk of Bias summary using Cochrane Risk of Bias Tool. **Figure S3.** Risk of Bias graph using Cochrane Risk of Bias Tool. Click here for file
