# Supplementary material for: Is group cognitive behaviour therapy for postnatal depression evidence-based practice? A systematic review
Source: BMC Psychiatry. 2013 Nov 28;13:321. doi: 10.1186/1471-244X-13-321 (PMC4219505; doi:10.1186/1471-244X-13-321)

Additional file

Figure S1: *Summary of study selection and exclusion*

Potentially relevant papers identified and screened for retrieval

Original search (n=7,633)

Update search (n=2,547)

Total abstracts screened

Original search (n=3,451)

Update search (n=186)

Total full papers screened

Original search (n=153)

Update search (n=55)

Studies potentially relevant

Original search (n=23)

Update search (n=7)

Studies excluded at title sift

Original search (n=4,182)

Update search (n=2,361)

Studies excluded at abstract sift

Original search (n=3,298)

Update search (n=131)

Studies excluded at full paper sift

Original search (n=130)

Update search (n=48)

Studies excluded on the basis of inclusion/exclusion criteria (employed CBT)

Original search (n=17)

Update search (n=6)

Total included full papers

Original search (n=6)

Update search (n=1)

(RCTs, n=3; Non RCTs, n=4)

Figure S2. *Risk of Bias summary using Cochrane Risk of Bias Tool*


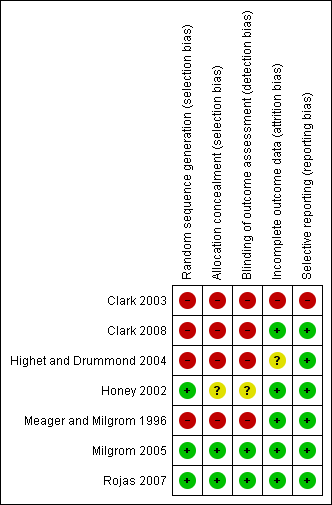


Figure S3. *Risk of Bias graph using Cochrane Risk of Bias Tool.*


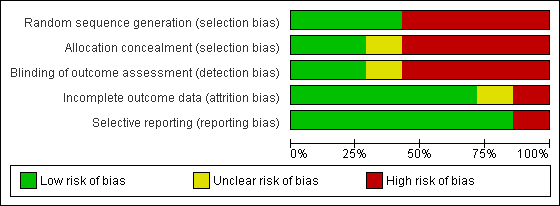

Supplement: Additional file 1: Figure S1 — Summary of study selection and exclusion. Figure S2. Risk of Bias summary using Cochrane Risk of Bias Tool. Figure S3. Risk of Bias graph using Cochrane Risk of Bias Tool. [file 1471-244X-13-321-S1.docx]
